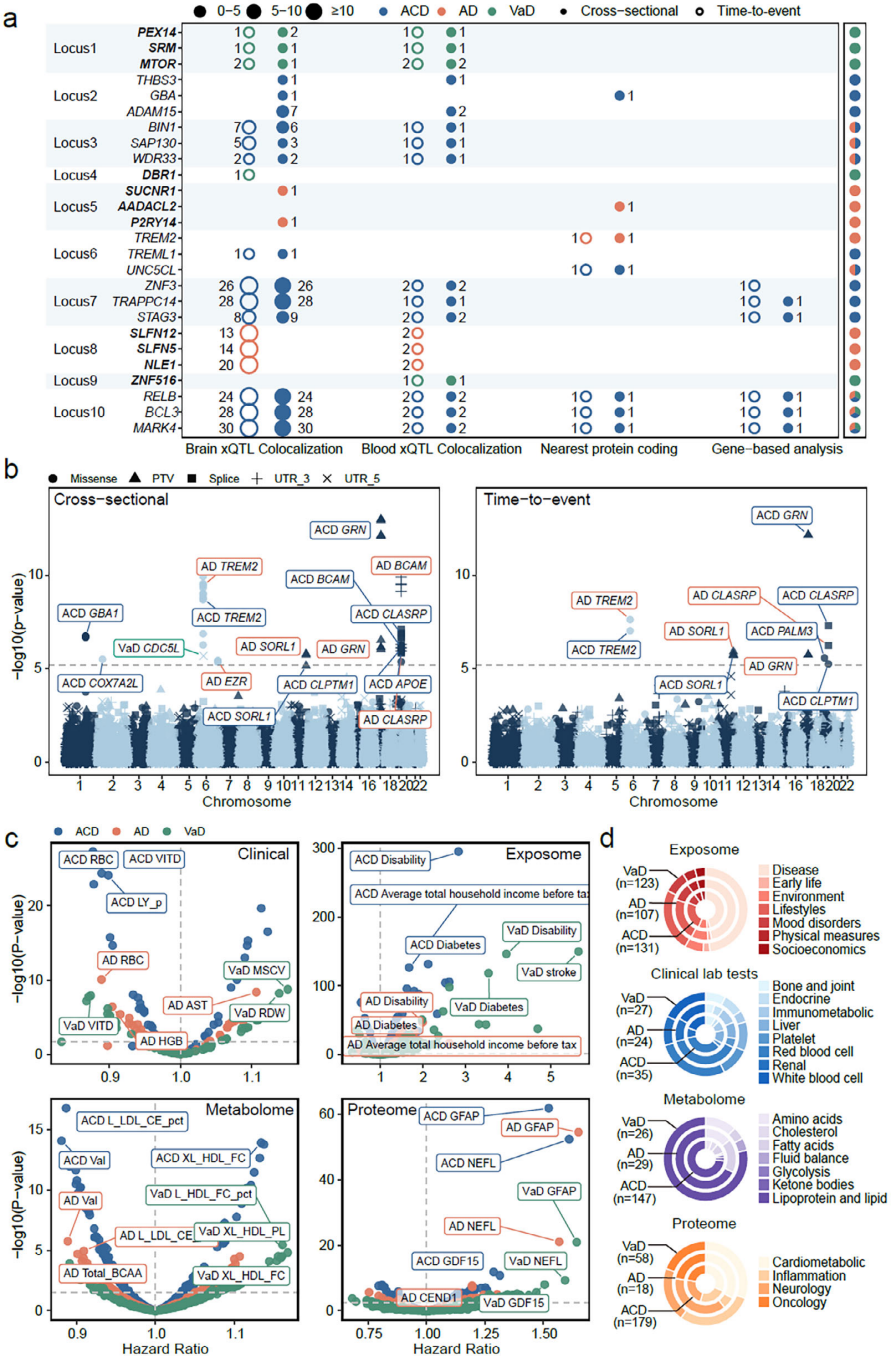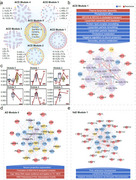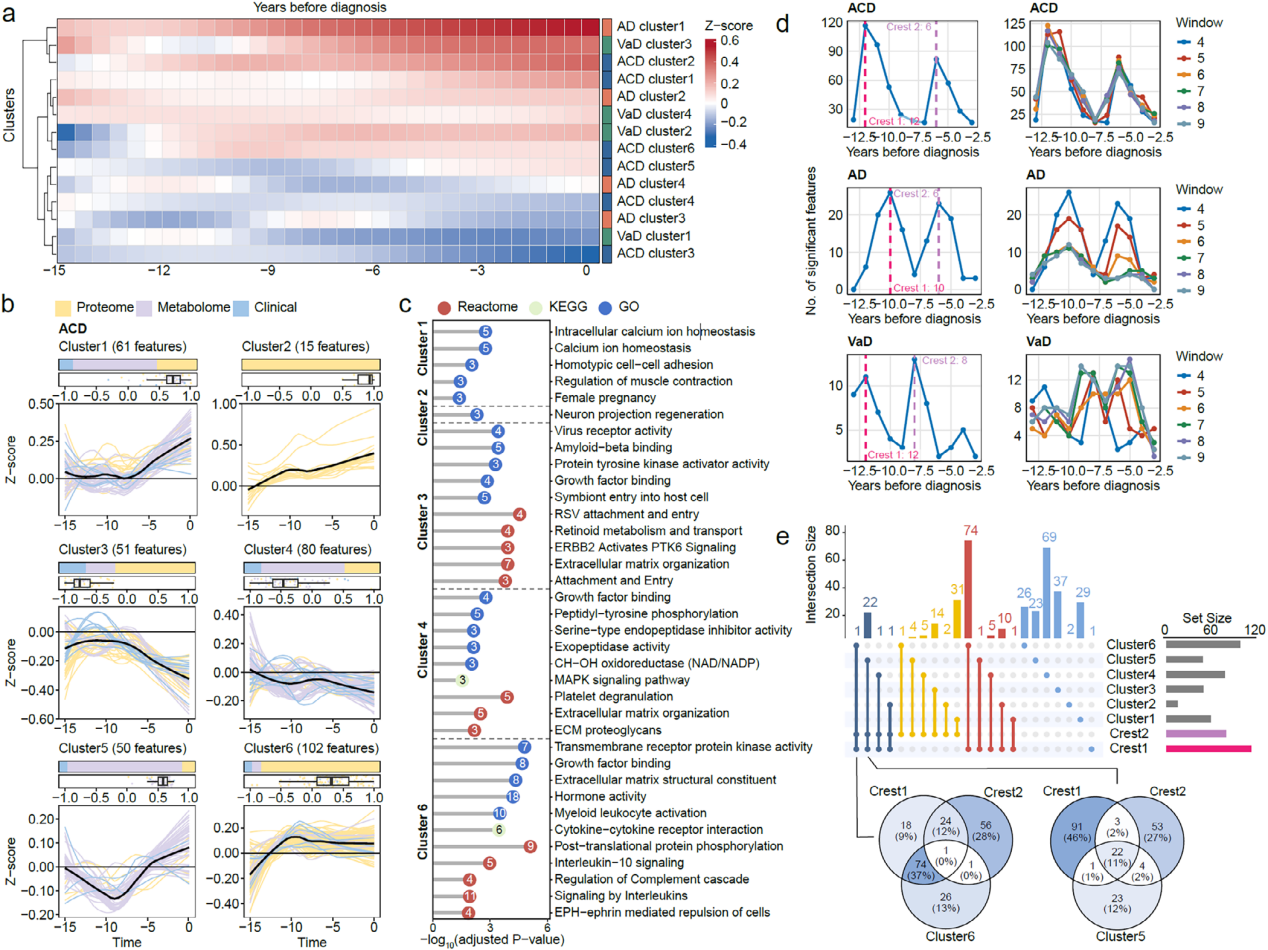# Integrated Multi‐Omics Analysis Reveals Molecular Mechanisms and Identifies Potential Therapeutic Targets for Dementia

**DOI:** 10.1002/alz70856_103240

**Published:** 2025-12-25

**Authors:** Xiao‐Yu He, Jin‐Tai Yu

**Affiliations:** ^1^ Huashan Hospital, Fudan University, Shanghai, China; ^2^ Huashan Hospital, Fudan University, Shanghai, Shanghai, China

## Abstract

**Background:**

Dementia, a leading cause of global disability, poses significant socioeconomic burdens. Early identification of biomarkers and understanding the molecular mechanisms underlying dementia are crucial for developing effective interventions. Multi‐omics approaches offer a comprehensive framework to unravel the complex interplay of genetic, proteomic, metabolomic, and clinical factors in dementia pathogenesis.

**Method:**

We conducted a longitudinal multi‐omics analysis integrating whole‐genome sequencing (WGS), proteomics, metabolomics, and clinical laboratory data from a large cohort (*N* = 320,226). Single‐omics association analyses were performed to identify dementia‐related features, followed by cross‐omics integration using advanced machine learning models. We employed mediation analysis and Mendelian randomization to assess causal relationships and identify potential drug targets and actionable antecedents.

**Result:**

We identified 203 lead genetic variants associated with all‐cause dementia (ACD), Alzheimer's disease (AD), and vascular dementia (VaD), implicating 228 candidate genes. Multi‐omics network analysis revealed distinct biological modules, such as lipid metabolism dysregulation and synaptic dysfunction in ACD. Longitudinal trajectory analysis demonstrated dynamic changes in blood multi‐omics profiles up to 15 years before dementia diagnosis, with specific clusters of proteins and metabolites showing early and sustained elevation. The FT‐Transformer model significantly improved dementia risk prediction by integrating multi‐omics data, outperforming single‐omics models. Mediation analysis identified 258 mediators, with proteins like GDF15, IGFBP2, and NEFL playing key roles in dementia risk. Furthermore, we identified 24 potential drug targets, including 8 proteins with existing drugs in clinical use or trials, and 7 novel targets with favorable safety profiles.

**Conclusion:**

This study provides a comprehensive multi‐omics framework for understanding the molecular mechanisms of dementia, identifying predictive biomarkers, and uncovering potential therapeutic targets. Our findings highlight the dynamic evolution of multi‐omics profiles before dementia onset and offer new avenues for early intervention and drug repurposing.